# Swallowing prehabilitation for people with head and neck cancer: a pilot cluster-randomised feasibility trial of the SIP SMART intervention

**DOI:** 10.1136/bmjopen-2025-103559

**Published:** 2025-09-25

**Authors:** Roganie Govender, Jiunn Wang, Louise Marston, Elena Pizzo, Stuart Taylor, Irwin Nazareth

**Affiliations:** 1 Head and Neck Academic Centre, Division of Surgery & Interventional Science, University College London, London, UK; 2 Head and Neck Cancer Centre, University College London Hospitals NHS Foundation Trust, London, UK; 3 Department of Primary Care and Population Health, University College London, London, UK; 4 University College London, London, UK; 5 Priment Clinical Trials unit, University College London, London, UK; 6 Centre for Medical Imaging, University College London Hospitals NHS Foundation Trust, London, UK

**Keywords:** Head & neck tumours, Preventive Health Services, Feasibility Studies

## Abstract

**Objectives:**

To assess the feasibility of delivering the swallowing prehabilitation intervention known as Swallowing Intervention Package: Self-Monitoring, Assessment and Rehabilitation Training (SIP SMART) within the National Health Service (NHS) head and neck cancer care pathway.

**Design:**

Two-arm cluster-randomised pilot trial: SIP SMART2 trial.

**Setting and participants:**

Adults newly diagnosed with stage II–IV head and neck cancer receiving curative treatment within a multidisciplinary team who agree to participate.

**Interventions:**

Six hospitals were randomised. Trained clinicians at the intervention sites delivered the manualised SIP SMART intervention, while standard care was provided at care as usual (CAU) sites. The intervention included two 45-minute consultations incorporating an X-ray swallow assessment, tailored exercises/advice and specific behaviour change strategies while CAU involved a single consultation of information giving and provision of a generic exercise sheet.

**Outcomes:**

Study outcomes related to feasibility of the cluster-randomised design, recruitment of both sites and patients and completeness of clinical and health economic data collected at baseline, 4 weeks, 12 weeks and 24 weeks after treatment.

**Results:**

12 hospitals expressed interest and six were randomised (50%) and provided data to the point of study completion. Patient recruitment across all sites (n=76) reached the target, although two sites fell short of their individual targets. The proportion of people with HNC recruited versus those eligible for each arm was 39% (95% CI 29 to 49) for SIP SMART group and 55% (95% CI 43 to 66) for CAU. The end point data at 24 weeks were completed for 50% (95% CI 33 to 67) for SIP SMART and 78% (95% CI 62 to 89) for CAU. Adherence to the intervention was above 50% at all time points. No harms related to the intervention were reported.

**Conclusions:**

It is feasible to deliver the SIP SMART intervention embedded within the NHS cancer care pathway using a cluster-randomised design. A future trial will be optimised for efficiency in set-up and follow-up data collection based on these findings and learnings from the accompanying process evaluation study.

**Trial registration number:**

ISRCTN12377415.

**STRENGTHS AND LIMITATIONS OF THIS STUDY**

- The cluster-randomised design allowed all speech and language therapists involved in pretreatment consultations at a site to receive the intervention training promoting participation and buy-in.
- The cluster design was useful to mitigate issues of contamination, particularly as it was not possible to blind staff or patients to the intervention.
- Unlike a parallel group randomised controlled trial (RCT) with individual patient randomisation, even one poorly performing site in a small cluster RCT can have a profound impact on the overall trial.
- With only six clusters randomised, this pilot study suffered from the generally poor follow-up at one site.

## Introduction

Head and neck cancers (HNCs) impinge on the vital structures within the mouth and throat involved in eating, drinking, swallowing, speaking and breathing. Dysphagia may be present before treatment^1^ and may persist in 45–70% of people after treatment completion.^2^,^4^ Survivors of HNC are left with high functional burden and consequent cost implications for the affected individuals and their families^5^ and, by extension, wider society.

Dysphagia can also lead to other health complications such as aspiration pneumonia, malnutrition and depressive symptoms; all are associated with further health and care service needs and increased cost implications.^6^ It may be possible to mitigate some of the ramifications of dysphagia through prehabilitation.^7^ Prehabilitation is recognised within the diet and physical exercise literature as a potential opportunity to improve outcomes in patients with cancer beyond what can be achieved by post-treatment rehabilitation alone.^8^ While the low quality and level of evidence for prophylactic swallow exercises remain a problem,^9 10^ several studies show that pretreatment swallowing exercises undertaken prior to the start of radiotherapy can improve postradiotherapy outcomes for eating, drinking and swallowing.^11^,^13^ The evidence to date is drawn mainly from studies that have focused on a specific protocol of exercises and have usually limited the sample to patients undergoing radiotherapy with/without adjuvant chemotherapy.^10 14^

Cancer prehabilitation, however, is more broadly characterised as encompassing psychological support, education and exercise provided prior to cancer treatment, including surgery.^15^ Accordingly, the intervention proposed in the current study was developed using this broader definition. Swallowing Intervention Package: Self-Monitoring, Assessment and Rehabilitation Training (SIP SMART) is a theory-based behaviour change intervention which aims to address dysphagia-related quality of life (QoL) after HNC treatment through education, counselling and tailored exercises/advice provided to patients prior to the start of cancer treatment involving any modality with curative intent. Cancer care in the English National Health Service (NHS) is usually delivered by multidisciplinary teams (MDTs) required to follow national guidelines of good practice. Thus, interventions aimed at improving post-treatment functional outcomes and QoL need to be specifically designed for and embedded into the NHS cancer care pathway from the outset. The rationale for and development of the SIP SMART intervention have been previously reported in a series of papers.^16^,^20^ A preliminary study of the feasibility of the intervention was tested at a single NHS hospital site using a small-scale parallel group RCT design.^21^ The preliminary results showed satisfactory feasibility and positive signals for the SIP SMART intervention on the clinical outcomes. However, the intervention was delivered by the first author (RG) who also led on the development work. A larger trial would require clinicians to receive training to deliver the intervention in other settings, meaning that it was necessary to avoid contamination and preserve equipoise if the same clinician was required to deliver both arms of the study at their hospital. Accordingly, this multicentre cluster-randomised pilot trial was undertaken to address key uncertainties and to inform refinements to the intervention and/or research process in preparation for a main trial. The overarching aim of a future definitive trial is to determine whether people with HNC who participate in a complex prehabilitation behaviour change intervention, tailored to educate, counsel and facilitate swallowing exercises/advice over and above routine usual care, achieve better dysphagia-related QoL at 6 months compared with usual care. The primary aim of the current external pilot trial (named SIP SMART2) was to examine the acceptability and feasibility of conducting the definitive trial using a cluster randomised design. In line with the primary goals of pilot and feasibility trials,^22^ our specific objectives were to assess the:

1. Proportion of hospitals invited who agree to participate in the RCT (test of sampling, recruitment and retention approaches for hospital sites).
2. Proportion of people with HNC approached at the study sites in both trial arms who consent to participation and agree to provide outcome data for research evaluation (patient recruitment).
3. Proportion of patients in the intervention and control groups for whom it is possible to collect follow-up data to the point of assessing the primary outcome (clinical effectiveness as determined by the MD Anderson Dysphagia Inventory^23^ score (MDADI) and data relevant for an economic evaluation.
4. Proportion of data present on each outcome at all data points measured in both arms.
5. Proportion of patients who report satisfactory adherence to the intervention as measured by an adherence questionnaire.

In addition, we conducted a process evaluation of factors related to context and implementation pre-trial, during trial and post-trial. These findings will be addressed in a separate paper focusing on qualitative data collected to improve efficiency of the future main trial and inform plans for any relevant mitigations.

## Methods

### Study design

The cluster randomised design was selected to avoid contamination, with the hospital/cancer centre as the unit. This was done as the speech and language therapists (SLTs) delivering the intervention received specific training in behaviour change techniques and were required to implement the SIP SMART manualised intervention to all eligible and consenting patients at their site. A pragmatic decision was taken to recruit six hospital sites providing multidisciplinary HNC care based on what was achievable within a fellowship funding award. Hospitals were randomised in a 1:1 ratio and allocated to ‘care as usual’ and ‘SIP SMART intervention’ groups. A randomisation list was created by a statistician who was independent of the RCT. All sites were randomised at the same time and notified of the outcome on the same day. The study statistician and health economist were blinded to the randomisation list. However, it was not possible to blind the study participants and those treating them due to the nature of the intervention.

### Patient and public involvement

Patients were involved in reviewing the patient information leaflets and producing a guide on top tips for approaching patients newly diagnosed with cancer. A patient representative was also appointed to the trial steering committee and participated in copresenting at a conference.

### Participants

Adults over 18 years, who presented with newly diagnosed stage II–IV head and neck squamous cell carcinoma of the oral cavity, oropharynx, nasopharynx, hypopharynx, mandible and maxilla were eligible. Tumours with an unknown primary, presenting with neck lump and undergoing radiotherapy were included. Patients must have been discussed at a multidisciplinary meeting and planned for treatment with curative intent via surgery, radiotherapy, chemoradiotherapy or combinations thereof. HNC treatment regimens at NHS centres are broadly consistent for tumour sites and staging.^24^

Patients with a previous diagnosis of HNC, those receiving palliation or being treated solely by non-standard treatment such as photodynamic therapy, brachytherapy or chemotherapy alone were not eligible. Patients who were planned for a total laryngectomy or long-term tracheostomy, those deemed vulnerable or who had significant comorbidities as determined by a score of 4 on the WHO performance status scale were also excluded. Finally, patients who had brain tumours and other primary tumours not within head and neck, and those unable to provide informed consent were excluded.

### Recruitment of sites

This was achieved via an open advert to NHS head and neck centres or hospitals in England who operate within an MDT. The advert was circulated via clinical excellence networks and meetings with attendance by key clinicians working with this patient population. For inclusion, sites were required to be willing to be randomised as a unit, have a minimum of two SLTs who were available to provide the SIP SMART intervention and were prepared to undergo 2–3 days of training. Sites needed access to videofluoroscopy services at least twice a week (for X-ray swallow assessment) and were required to have regular MDT meetings at which new cases of HNC were discussed. All sites that expressed an interest were screened using a site screening form and the first six sites that met the criteria, agreed and consented to participation were randomised.

### Recruitment of patients

Potential patients were identified from the new referrals received by the head and neck MDT at each site. Prescreening was done by appropriately trained clinicians or research nurses based on the list of new patients with eligible diagnoses circulated to the MDT members prior to the multidisciplinary discussion. In this way, potentially eligible patients flagged for the SIP SMART2 study were highlighted for discussion and recorded on the research screening log. In most centres, the consultant and other members of the clinical team met the patient at the afternoon clinic to discuss a treatment plan. At this point, if appropriate, the patient would be informed about the study and invited to participate. The research nurse or principal investigator was asked to screen the patient for eligibility and to offer further information about the study and a copy of the patient information leaflet, allowing at least 24 hours prior to seeking/taking consent. Members of the clinical team, including dedicated research nurses or facilitators, were responsible for screening and recruiting participants.

### Standard care group

Patients recruited at sites randomised to CAU received the standard care offered by the SLT service. A pretrial meeting of clinicians at all CAU sites helped to ensure that key elements of usual care were broadly consistent and documented. CAU included a baseline clinical assessment of swallowing, information about the upcoming treatment and its impact on swallowing, as well as the provision of a generic swallowing exercise leaflet. CAU was delivered in one consultation and normally with other members of the team including a dietitian and clinical nurse specialist. A usual care manual devised during the preliminary study of SIP SMART was available and could be adapted for use at CAU sites.

### Intervention group

Patients in the intervention group received the manualised SIP SMART intervention. The intervention was designed to take place over two 45 min consultations. These could occur in succession on the same day or with a day or two between them, depending on patient preference. The new intervention was delivered by SLTs who completed a bespoke 2 day training course in behavioural counselling delivered by externally commissioned trainers with experience in behaviour change interventions in healthcare. The SIP SMART intervention additionally included the following:

- Patients underwent an X-ray assessment of their swallow function (modified barium swallow, MBS) in the fluoroscopy suite at the hospital site. This procedure was part of the SIP SMART intervention informing the selection of targeted exercises and was used as part of patient education. A standard protocol for this clinical procedure was adopted for the study.^25^ The recording was available for later analysis of swallow biomechanics and selection of appropriate targets for exercises by the local SLT.
- Patients were subsequently shown a video-animation (Dysphagia App, Northern Speech Services, Michigan, USA) to explain the basic mechanics of swallowing and to orientate them to key structures such as the tongue, base of tongue, airway and oesophagus. Patients were thereafter shown a recording of their own MBS examination and helped to identify the key structures using this newly acquired knowledge. The SLT encouraged patients to provide commentary and/or ask questions as they watched the video, a key part of the SIP SMART intervention described in prior work.^19^
- The MBS assessment was used to tailor the information, advice and exercises given to the patient during the pretreatment session and to facilitate discussion about the rationale for advice and exercises and possible consequences of not doing exercises.
- SLTs were trained in behavioural strategies to be used throughout the consultation to promote good adherence to the advice and exercises provided.

Each SLT delivering the intervention was required to audio record at least one consultation which would allow for fidelity assessment, while patient adherence was reported using a study specific self-reported questionnaire.

Following the oncological treatment (surgery or radiotherapy), patients in both groups followed the usual care pathway for SLT rehabilitation post treatment. The number of SLT rehabilitation sessions for all patients was recorded. Patients were informed that the advice and exercises may be amended after treatment based on updated assessments.

### Outcomes

Primary outcomes reflect the stated feasibility questions. Table 1 illustrates a summary of these along with progression criteria using the traffic light system. Thresholds were agreed by the trial management group using information from the literature and knowledge of the practical context. It was envisaged that these criteria would be evaluated in combination when deciding on feasibility.

**Table 1 T1:** Outcomes for this feasibility study with prespecified criteria to inform progression to main trial

Criterion	Assessment method	Go – proceed to main trial	Amend – proceed with changes	Stop – do not proceed unless changes are possible
Proportion of hospitals who agreed to participate	Test of sampling, recruitment and retention approaches for sites	80% (5/6 sites)	66% (4/6)	50% (3/6 or less)
12 patients recruited at each site over 6–8 months*	Screening logs	5/6 sites achieve target	4/6 sites achieve target	3/6 or less
Proportion of patients in both groups for whom it is possible to collect follow-up data to the point of primary outcome (clinical effectiveness-MDADI)	Assessment of data completeness	>70% for MDADI	60–70%	<60% Consider factors from process evaluation for example, use of iPad, postal, remote data collection and how this influences follow-up. Presence of research nurse support.

* 12 was considered a feasible target based on disease prevalence and average numbers treated per annum at UK cancer centres.

MDADI, MD Anderson Dysphagia Inventory.

Secondary outcomes included the clinical measures for dysphagia and QoL, costs, as well as health economics outcomes. The Schedule of assessments and collection of follow-up outcome measures is shown in table 2.

**Table 2:** Schedule of assessments and timing of all data collection points

Visit no	Baseline	Intervention	4-week follow-up	12-week follow-up	24-week follow-up
Window of flexibility for timing of visits: from last oncological treatment (day of surgery or last day of radiotherapy)			±7 days	±7 days	±7 days
Informed consent	X				
Medical history: including smoking and alcohol use via the AUDIT-C tool (https://www.gov.uk/government/publications/alcohol-use-screening-tests/guidance-on-the-5-alcohol-use-screening-tests) where a score of 5 or more indicates increasing risk of alcohol-related harm	X				
Physical/oral peripheral examination: including Functional Intra-oral Glasgow Scale^44^ which provides a clinician rating of swallow, speech, chewing rated from 1 (poor function) to 5 (optimal function)	X				
Weight and feeding tube status	X		X	X	X
Eligibility confirmation	X				
WHO performance status scale Performance of everyday tasks with 0 being normal and 4 unable to self-care	X				
Performance Status Scale-Head and Neck^45^ Measure of normalcy of diet and social eating Rated by clinician from 0 (poor function) to 100 (optimal function)	X		X	X	X
Maximum incisor opening measure of jaw opening, where less than 35 mm is considered restrictive for normal functioning	X		X	X	X
100 mL water swallow test^46^ number of swallows and number of seconds taken to drink 100 mL water used as a clinical measure of swallow ability	X		X	X	X
MD Anderson Dysphagia Inventory^23^ A dysphagia related QoL questionnaire with composite scores from 0 to 100 where higher scores reflect better QoL	X		X	X	X
Functional Assessment of Cancer Therapy- Head and Neck, www.facit.org Overall health-related QoL after cancer ranging from 0 to 144 with higher scores indicating better QoL	X		X	X	X
Euro Quality of Life, www.euroqol.org Non-disease specific tool to measure health related QoL used in health economics assessment, −0.59 to 1 with 1 being best health	X		X	X	X
UK cancer cost questionnaire, www.dirum.org	X		X	X	X
Self-reported adherence questionnaire			X	X	X
Trial intervention/treatment		X			
Adverse events review	X	X	X	X	X
Concomitant medication review	X	X	X	X	X
Withdrawal	X	X	X	X	X

AUDIT-C, Alcohol Use Disorders Identification Test-Consumption; QoL, quality of life.

### Statistical analysis

The analysis and reporting conform to the Consolidated Standards of Reporting Trials (CONSORT) extension to randomised pilot and feasibility trials.^26^ All data were described overall and by randomised group using descriptive statistics. We did a baseline predictor of missingness analysis using logistic regression with robust SEs to account for clustering by site for MDADI and Functional Assessment of Cancer Therapy-Head and Neck (FACT-HN) at 24 weeks. This analysis was planned to use mixed effects logistic regression, but this did not converge due to small numbers in clusters and/or perfect prediction.

To gather relevant data for a future cost-effectiveness study, we undertook a preliminary health economic analysis from both the NHS and the societal perspectives. To calculate costs, we used an adapted UK Cancer Costs Questionnaire to capture the NHS resource use and then applied unit costs derived from the national references for each patient (See online supplemental tables 2–5). Health economic outcomes were FACT-HN and quality-adjusted life years (QALYs), estimated using Euro Quality of Life questionnaire. Assuming a linear trajectory over time, outcomes were calculated as the area under the curve. Ordinary least squares regressions were applied to examine differences in costs and outcomes between groups. As this analysis is preliminary, these results are presented in online supplemental tables 2–6.

## Results

**Figure 1:**
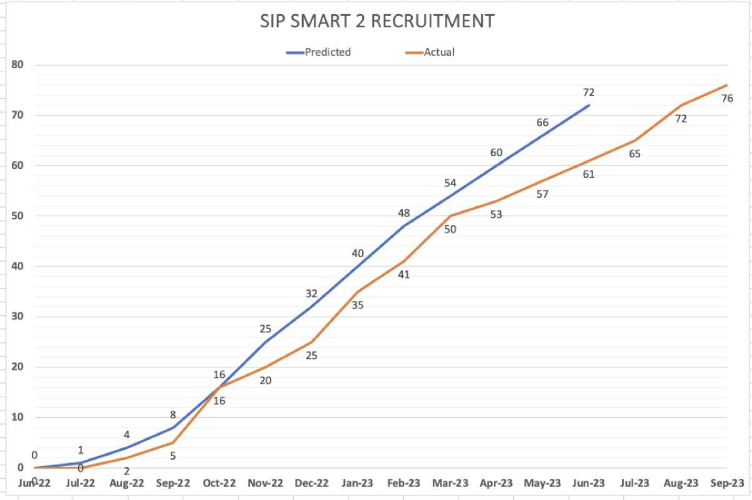
Predicted and actual accrual of participants into the trial over the recruitment timeframe. SIP SMART, Swallowing Intervention Package: Self-Monitoring, Assessment and Rehabilitation Training.

**Figure 2:**
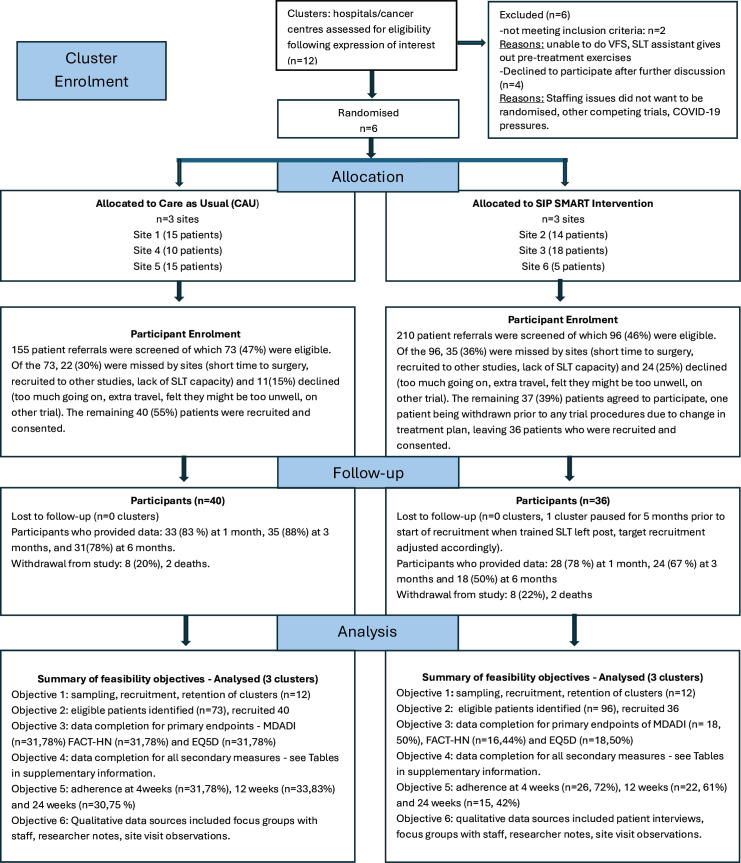
Consolidated Standards of Reporting Trials flowchart adapted for cluster-randomised feasibility trial. CAU, care as usual; EQ5D, Euro Quality of Life; FACT-HN, Functional Assessment of Cancer Therapy-Head and Neck; MDADI, MD Anderson Dysphagia Inventory; SIP SMART, Swallowing Intervention Package: Self-Monitoring, Assessment and Rehabilitation Training; SLT, speech and language therapist; VFS, videofluoroscopy.

This study was affected by the COVID-19 pandemic in terms of set-up, recruitment timeframe and staffing resources. Despite these challenges, target recruitment was achieved for the number of sites randomised and the overall patient numbers recruited across sites. Recruitment took place between June 2022 and September 2023, with unavoidable delays in site activation at some centres. All sites received their randomisation allocation and site information pack on the same day, but the time lag to sites being activated for recruitment varied considerably, mainly due to local research and development (R&D) delays. Clinician training took place within 4 weeks of randomisation. While the planned timeline was to aim for all sites to be opened within 90 days, this was not achieved. The mean time from site randomisation to recruitment of the first patient in each arm was: intervention—340 calendar days (range, 166–461) and care as usual—148 calendar days (range 111–186). The final 24-week follow-up of the last patient (from date of final oncological treatment) was completed in June 2024. Figure 1 illustrates the overall recruitment rate, while figure 2 is the CONSORT flowchart showing the recruitment of sites and patient numbers achieved at each site separated by allocation group.

Table 3 presents the baseline characteristics of patients recruited in each arm of the trial with similarity between groups for most features. Swallowing measures at baseline are also similar and usually within good to normal values as expected. People in the SIP SMART group had a higher baseline mean weight and body mass index. This group also had fewer current smokers and more ex-smokers compared with the CAU group. Also, the percentage of people divorced was almost four times more for CAU compared with SIP SMART, while the number of people educated to degree level or higher was about 3.6 times more in the CAU group. Co-incidentally, the randomisation process resulted in the intervention clusters all falling in the north and the CAU clusters in the south of the country. These findings are expanded on in the Discussion section.

**Table 3:** Patient characteristics and swallow status at baseline

Characteristic	SIP SMART		Treatment as usual	
	n/N or mean	% or (SD)	n/N or mean	% or (SD)
Male	25/36	69	27/40	68
Age	62.3	(9.1)	63.9	(9.4)
Ethnicity				
White	33/36	91.67	37/40	92.50
Mixed/multiple ethnic groups	1/36	2.78	1/40	2.50
Asian/Asian British	1/36	2.78	2/40	5.00
Chinese	1/36	2.78	0/40	0.00
Marital status				
Married/ cohabiting	20/36	56	22/40	55
Divorced	2/36	6	9/40	23
Widowed	11/36	31	7/40	18
Single/separated	3/36	8	2/40	5
Highest education				
Secondary	18	54.55	11	30.56
Specific vocational training	3	9.09	3	8.33
Other vocational training	4	12.12	6	16.67
University degree (undergraduate)	2	6.06	7	19.44
University degree (postgraduate)	2	6.06	9	25.00
Other general education	4	12.12	0	0.00
Missing	3		4	
Employment				
Employment (full time)	9/36	25.00	13/40	32.50
Employment (part time)	3/36	8.33	2/40	5.00
Self-employed	3/36	8.33	7/40	17.50
Unemployed	0/36	0.00	2/40	5.00
Retired	18/36	50.00	15/40	37.50
Long-term sick leave	3/36	8.33	1/40	2.50
Weight	86.1	(14.1)	77.9	(20.2)
Height	1.73	(0.09)	1.71	(0.09)
Body mass index	28.8	(5.0)	26.4	(5.6)
Current smoker	5/34	15	11/40	28
Quit in the last 6 months	4/34	12	1/40	3
Quit more than 6 months ago	13/34	38	13/40	33
Never smoked	12/34	35	15/40	38
Missing	2			
AUDIT-C score	4	(4)	5	(3)
Tumour staging (AJCC v.8)				
I	5/34	15	10/40	25
II	17/34	50	11/40	28
III	12/34	35	19/40	48
Missing	2			
100 mL water swallow test				
Number of swallows	5	(2)	6	(3)
Time to finish (seconds) median (IQR)	7	(6, 9)	7	(5, 8)
Amount swallowed median (IQR)	100	(100, 100)	100	(100, 100)
Signs of aspiration/penetration	4/35	11	5/38	13
Volume	21.3	(8.7)	21.2	(10.8)
Capacity	15.0	(6.7)	15.5	(7.4)
Speed median (IQR)	1.3	(1.1, 1.7)	1.3	(1.1, 1.6)
Performance Status Scale-Head and Neck (0–100)				
Normalcy of diet median (IQR)	100	(50, 100)	100	(60, 100)
Public eating median (IQR)	100	(75, 100)	100	(100, 100)
Understandability of speech median (IQR)	100	(100, 100)	100	(100, 100)
Maximal incisor opening (mm)	47	(12)	43	(9)
Functional Intra-oral Glasgow Scale (FIGS) (0–5)				
Speech median (IQR)	5	(5, 5)	5	(5, 5)
Chewing median (IQR)	5	(3, 5)	5	(4, 5)
Swallowing median (IQR)	5	(4, 5)	5	(4, 5)
Overall FIGS score median (IQR)	15	(12, 15)	14	(13, 15)
Functional Assessment of Cancer Therapy-Head and Neck total score (0–144)	116	(23)	114	(18)
MD Anderson Dysphagia Inventory Composite Score (0–100)	81	(13)	83	(11)
Euro Quality of Life (−0.59 to 1)	0.74	(0.2)	0.76	(0.2)
Radiotherapy received	31/34	91	35/38	92
Chemotherapy received	22/34	65	20/38	53
Surgery received	8/34	24	16/38	42
Treatment not completed/missing	2		2	

Variable denominators reflect total number with complete data for specific characteristic.

AUDIT-C, Alcohol Use Disorders Identification Test-Consumption; SIP SMART, Swallowing Intervention Package: Self-Monitoring, Assessment and Rehabilitation Training.

### Overall feasibility outcomes

#### Site recruitment and retention

As shown in figure 2, 12 sites expressed interest in participating prior to the close of advert and half of these were recruited, consented and randomised. All six sites remained in the trial to the end and contributed data. One site paused the study for 6 months when the key SLT delivering the intervention left her post. The target recruitment number for the site was subsequently halved (in discussion with the trial steering committee) to support the site to remain in the trial with a new SLT being trained. The overall recruitment timeframe was also increased by 3 months so that all sites had a 6-month window for recruitment from the date of site activation. Site recruitment and retention therefore met the green ‘Go’ criterion, suggesting that the methods for recruiting sites were adequate. This was even though many sites expressed reluctance due to the COVID-19 pressures and the fact that reasonable adjustments had to be made to allow for administrative delays in opening sites.

#### Patient recruitment

The total number of patients screened for this study across all sites was 365 and the total number eligible was 169 (46%). Of those eligible, 76 patients (45%) consented and were recruited into the study. Importantly, 34% were missed for reasons other than patient declining to participate, which was about a fifth of those eligible (21%). Following the agreed adjustment in target numbers for site 6, only one other site did not achieve the minimum target of 12 patients. All other sites exceeded the target within the recruitment window. Figure 2 displays this information for each trial arm.

### Feasibility outcomes by randomised group

Feasibility outcomes for each arm of the trial are also illustrated in figure 2. In both groups, all sites that signed up contributed to data collection, although the proportion of people recruited relative to the number eligible was higher in the CAU group (55%) compared with the intervention group (39%). Despite this, in both arms, two of the three sites achieved the initial minimum target of 12 patients. Data completion for the primary endpoint was less satisfactory with only 50% completion in the intervention group and 78% in the CAU group. Patient reported adherence to the received intervention was above the 50% threshold at all time points. Detailed tables of the amount of data present by randomised group at each data point are available in online supplemental table 1 [Table T1]. When looking at predictors of missingness, group allocation was paramount. There was a 70% lower chance of missing data if allocated to the CAU group. Data were severely skewed by allocation to the SIP SMART group primarily driven by the extremely low completion of follow-up outcomes at site 2.

### Adverse events and withdrawals by randomised group

More adverse events were reported for the CAU group (n=10) than the intervention group (n=1). Aside from deaths, six other SAEs were reported in the CAU group and one in the SIP SMART group, all relating to hospital admissions primarily for a feeding tube during treatment. As these were all expected and resolved, they were subsequently downgraded to adverse events in line with the protocol. The number of deaths and withdrawals was similar in both arms: both had eight withdrawals, including two deaths in each, resulting in a 21% (16/76) attrition overall. The deaths were from disease recurrence or other causes unrelated to the study intervention. Withdrawals were mainly due to patients moving away from the treating centre or not responding to repeated contact from the study team. No safety issues were reported that were deemed attributable to the intervention.

### Secondary outcome measures

Data collected for clinical outcomes and cost utility show that baseline scores were comparable between groups (see table 3). Clinical outcomes obtained for SIP SMART versus CAU, respectively, were as follows: mean scores for maximum incisor opening (MIO) score=43 mm vs 41 mm; MDADI composite scores=67 vs 66; Performance Status Scale normalcy of diet=55 vs 50 and FACT-HN=106 vs 104.

### Health economics

The intervention costs were £143 for the CAU arm and £475 for the SIP SMART arm (see online supplemental tables 2–5). The increased cost from the SIP SMART arm is from the increased labour time of SLT over and above CAU and an additional X-ray assessment of the swallow function in the fluoroscopy suite at the hospital site. The preliminary health economics findings may be viewed in online supplemental table 6.

## Discussion

This pilot cluster-randomised trial of a swallowing prehabilitation intervention demonstrated that it was feasible to recruit the required number of hospitals as clusters even amidst the challenges of the global COVID-19 pandemic and the negative impact on staff and time resources. Patient recruitment also reached the overall target, although with adjustments in the timelines for opening sites to recruitment which inevitably increased the duration of the trial. It was reassuring to note that average adherence to the intervention was satisfactory (at least 50% of the time) or better. While all sites randomised remained in the trial and contributed data, the proportion of data present across both arms declined from baseline to the 6-month endpoint, partly due to attrition (deaths and withdrawals) and partly through failure to follow-up by sites resulting in missing data. Despite this, the data collected were sufficient to gather some early indications of the direction of effect. While the study was not designed to test effectiveness, and no significance testing was conducted, the clinical outcomes were almost all marginally better in the SIP SMART group compared with CAU. Positive trends for the efficacy of the new intervention may be drawn from these marginally better scores for dysphagia-related QoL (MDADI) at 6 months, the FACT-HN, the diet texture rating on the PSS – normalcy of diet and the MIO score. While very preliminary, SIP SMART shows good indicators to be cost-effective from both an NHS and societal perspective, based on FACT-HN.

Results from this current pilot study affirm and extend findings from the initial preliminary testing of SIP SMART^21^ undertaken to assess the acceptability of the intervention to patients and the feasibility of the trial processes at a single NHS site. Both the preliminary and pilot studies provide positive indicators for this theory-based intervention developed using the Medical Research Council guidelines^27^ for complex interventions and the Behaviour Change Wheel.^28^ Like swallowing prehabilitation RCTs conducted in Denmark,^29 30^ the Netherlands^11^ and the USA,^12 31 32^ the potential short to medium term benefit of prophylactic exercises on post-treatment clinical swallow measures and QoL is also noted in SIP SMART. The protocol for one other UK-based non-randomised feasibility study of a swallowing intervention preradiotherapy has been published.^33^ However, this study did not progress beyond feasibility, with very poor uptake of the electronic swallowing intervention package reported.^34^

While all prior RCTs report declining adherence over time and high drop-out rates of 25%–49%^10^ interestingly, satisfactory adherence (greater than 50% of the time) is reported by almost two-thirds of patients in both arms of the SIP SMART trial at 24 weeks. The challenge of patient self-reported adherence is, however, acknowledged. Self-reports are often overestimated when compared with wearable devices that directly monitor exercise adherence.^35^ The SIP SMART intervention builds in specific behaviour change techniques for self-monitoring^36^ which promotes more reliable adherence reporting through greater awareness and self-regulation.^37 38^ Such techniques were not included in the CAU group, which could therefore have been more influenced (inflated) by factors such as recall or wishing to ‘please’ the clinician. Notwithstanding the challenges of measuring patient-reported adherence, it is possible that the more holistic approach to prehabilitation (beyond providing prophylactic exercises) and the inclusion of behaviour change strategies could be shifting the adherence needle. As addressed in the development phase of the SIP SMART intervention,^17^ the underlying mechanisms by which such prehabilitation interventions work may therefore be even more important to understand during their design and implementation and to observe how this impacts clinical outcomes in the short to medium and long term post-treatment.

In addition to observing trends for clinical efficacy from our pilot data, we also sought to obtain preliminary indication of cost savings, which suggest that SIP SMART appears favourable from both NHS and societal perspectives. When using FACT-HN as the outcome measure, SIP SMART seems to show promise, although we recognise that these findings are tentative within the context of this pilot study. A publication from the Netherlands modelled on data from two swallowing preventative exercise studies demonstrated improvements in QALYs and showed that the preventative exercise intervention was more cost-effective than care as usual.^39^ Similarly, data from the ^40^USA and Canada^5^ also indicate the potential cost-effectiveness of pretreatment swallowing interventions compared with traditional post-treatment rehabilitation. We recognise that there are differences in healthcare systems, how care is delivered and the nuances of the interventions in different contexts. However, these international studies, along with our tentative findings, lend support for the potential cost-effectiveness of SIP SMART in a future trial.

### Strengths, limitations and key learning for a future trial

This pilot study benefitted from the foundational intervention development work for SIP SMART informed by theory and original research studies. Additionally, as the primary researcher is a clinical-academic embedded within the clinical specialty, there was a good basis for understanding the execution of the intervention and the trial processes within the context of NHS hospital settings. Furthermore, the opportunity to road-test the intervention content in a small-scale preliminary RCT at one centre provided valuable insights to progress to the current multicentre pilot trial where the focus was on testing the cluster-randomised design. An important strength in this trial is the fact that care as usual is broadly standardised by the strong community of practice of SLTs working in HNC and the availability of UK National Guidelines for HNC care.^24^

There were also challenges and limitations. First, there were significant amount of missing data with very few patients followed up at one site (site 2) randomised to the intervention arm. To reduce the amount of missing data, a larger trial with increased funding could employ strategies such as online questionnaires with automatic reminders and incentivisation, such as shopping vouchers for completion of all measures, as well as costing in more staff resources dedicated to collection of outcomes. In a definitive trial, it will also be appropriate to consider statistical or imputation methods for handling missing data.

Second, one site (site 6) was impacted by staff changes reducing the number of trained SLTs who could deliver the intervention. The study was paused at the site for a few months, resulting in a lower target recruitment being set as a compromise and as agreed by the trial steering committee. Unlike a parallel group trial, the cluster design suffers considerably from such scenarios, as it is not possible to simply replace the one site which experienced staff shortages as sites need to be randomised in pairs to preserve the integrity of the cluster design. This, of course, requires considerable additional set-up and training costs. In a future trial, it may be better to randomise in blocks and at slightly staggered time points, allowing more time for the accrual of more sites. This will also need to be balanced with how and when intervention training is offered, as this needs to occur after randomisation.

Third, although random, the intervention sites (clusters) were all in the north of the country and CAU in the south, with small variation of baseline characteristics observed that may have introduced regional bias of population demographics.^41^ Here too, methods of site accrual (including stratification) and randomisation will need to be carefully considered in a larger trial to mitigate the impact of regional/geographic clustering on trial end-points.

Finally, it was noted that a third of eligible patients were missed as they were not approached in a timely manner before the start of their cancer treatment. Given the small window for approaching patients, a future trial could maximise recruitment by ensuring proactive steps such as early conversations with sites to implement local strategies to address this potential issue. In this trial, the patient and public involvement (PPI) member was asked their opinion to troubleshoot issues in recruitment a few months into the trial. She devised a ‘tip sheet’ for approaching patients newly diagnosed with cancer that was circulated to sites. Positive feedback on its use was received from trial sites. Further refinements for a future trial that focus on context and implementation will be discussed in our forthcoming qualitative paper.

## Conclusion

Swallowing prehabilitation is worthwhile^42^ and has been shown here to be feasible to slot into the NHS care pathway in the window between diagnosis and the start of treatment. Findings from the recent Getting It Right First Time report^43^ show that dedicated prehabilitation is only currently taking place in 20% of the HNC networks in England, meaning that this is a priority area to address. There is potential for a swallowing prehabilitation intervention alongside the nutrition and physical activity programmes. However, evidence of clinical and cost effectiveness is an important first step in commissioning such an intervention. The current pilot trial of the SIP SMART intervention has met the key feasibility criteria when considered in combination, for recruitment of clusters and patients, although some aspects such as ensuring better follow-up data completion will need to be optimised for the future trial.

## Supplementary material

10.1136/bmjopen-2025-103559 online supplemental file 1

10.1136/bmjopen-2025-103559 online supplemental file 2

## Data Availability

Data are available upon reasonable request. All data relevant to the study are included in the article or uploaded as supplementary information.
