# Supplementary material for: Swallowing prehabilitation for people with head and neck cancer: a pilot cluster-randomised feasibility trial of the SIP SMART intervention
Source: BMJ Open. 2025 Sep 25;15(9):e103559. doi: 10.1136/bmjopen-2025-103559 (PMC12481345; doi:10.1136/bmjopen-2025-103559)
Supplement: online supplemental file 1 [file bmjopen-15-9-s001.docx]

**SUPPLEMENTARY INFORMATION**

**Supplementary Table 1: Proportion of data present at each data point for both arms of the trial**

| Characteristic | SIP SMART | | Treatment as usual | |
| --- | --- | --- | --- | --- |
|  | n/N | % (95%CI) | n/N | % (95%CI) |
| **Baseline** |  |  |  |  |
| Sex | 36/36 | 100 (90, 100) | 40/40 | 100 (91, 100) |
| Age | 34/36 | 94 (81, 99) | 36/40 | 90 (76, 97) |
| Ethnicity | 36/36 | 100 (90, 100) | 40/40 | 100 (91, 100) |
| Marital status | 36/36 | 100 (90, 100) | 40/40 | 100 (91, 100) |
| Highest education | 33/36 | 92 (78, 98) | 36/40 | 90 (76, 97) |
| Employment status | 36/36 | 100 (90, 100) | 40/40 | 100 (91, 100) |
| Weight | 30/36 | 83 (67, 94) | 40/40 | 100 (91, 100) |
| Smoking status | 34/36 | 94 (81, 99) | 40/40 | 100 (91, 100) |
| AUDIT-C score | 25/36 | 69 (52, 84) | 40/40 | 100 (91, 100) |
| Oro-motor/general clinical exam | 33/36 | 92 (78, 98) | 40/40 | 100 (91, 100) |
| Tumour staging | 34/36 | 94 (81, 99) | 40/40 | 100 (91, 100) |
| 100mL water swallow test | 35/36 | 97 (85,100) | 38/40 | 95 (83, 99) |
| PSS-HN | 35/36 | 97 (85,100) | 40/40 | 100 (91, 100) |
| Maximal incisor opening (mm) | 31/36 | 86 (71, 95) | 39/40 | 98 (87, 100) |
| Functional Intraoral Glasgow Scale | 35/36 | 97(85,100) | 40/40 | 100 (91, 100) |
| FACT-H&N total score | 25/36 | 69 (52, 84) | 39/40 | 98 (87, 100) |
| MDADI Composite Score | 34/36 | 94 (81, 99) | 40/40 | 100 (91, 100) |
| EQ-5D-5L index score | 25/36 | 69 (52, 84) | 40/40 | 100 (91,100) |
| **4-weeks** |  |  |  |  |
| Weight | 26/36 | 72 (55, 86) | 32/40 | 80 (64, 91) |
| 100mL water swallow test | 26/36 | 72 (55, 86) | 25/40 | 63 (46, 77) |
| PSS-HN | 26/36 | 72 (55, 86) | 32/40 | 80 (64, 91) |
| Maximal incisor opening (mm) | 25/36 | 69 (52, 84) | 28/40 | 70 (53, 83) |
| Functional Intraoral Glasgow Scale | 27/36 | 75 (58, 88) | 32/40 | 80 (64, 91) |
| FACT-H&N total score | 22/36 | 61 (43, 77) | 32/40 | 80 (64, 91) |
| MDADI Composite Score | 25/36 | 69 (52, 84) | 32/40 | 80 (64, 91) |
| EQ5-D-5L index score | 23/36 | 64 (46, 79) | 33/40 | 83 (67, 93) |
| Adherence questionnaire | 26/36 | 72 (55, 86) | 31/40 | 78 (62, 89) |
| **12-weeks** |  |  |  |  |
| Weight | 24/36 | 67 (49, 81) | 32/40 | 80 (64, 91) |
| 100mL water swallow test | 22/36 | 61 (43, 77) | 31/40 | 78 (62, 89) |
| PSS-HN | 24/36 | 67 (49, 81) | 35/40 | 88 (73, 96) |
| Maximal incisor opening (mm) | 21/36 | 58 (41, 74) | 27/40 | 68 (51, 81) |
| Functional Intraoral Glasgow Scale | 24/36 | 67 (49, 81) | 34/40 | 85 (70, 94) |
| FACT-H&N total score | 20/36 | 56 (38, 72) | 34/40 | 85 (70, 94) |
| MDADI Composite Score | 23/36 | 64 (46, 79) | 34/40 | 85 (70, 94) |
| EQ-5D-5L index score | 21/36 | 58 (41, 74) | 33/40 | 83 (67, 93) |
| Adherence questionnaire | 22/36 | 61 (43, 77) | 33/40 | 83 (67, 93) |
| **24-weeks** |  |  |  |  |
| Weight | 16/36 | 44 (28, 62) | 29/40 | 73 (56, 85) |
| 100mL water swallow test | 16/36 | 44 (28, 62) | 28/40 | 70 (53, 83) |
| PSS-HN | 16/36 | 44 (28, 62) | 31/40 | 78 (62, 89) |
| Maximal incisor opening (mm) | 13/36 | 36 (21, 54) | 28/40 | 70 (53, 83) |
| Functional Intraoral Glasgow Scale | 16/36 | 44 (28, 62) | 31/40 | 78 (62, 89) |
| FACT-H&N total score | 16/36 | 44 (28, 62) | 31/40 | 78 (62, 89) |
| MDADI Composite Score | 18/36 | 50 (33, 67) | 31/40 | 78 (62, 89) |
| EQ-5D-5L | 18/36 | 50 (33, 67) | 32/40 | 80 (64, 91) |
| Adherence questionnaire | 15/36 | 42 (26, 59) | 30/40 | 75 (59, 87) |
| Feeding tube (any time) over follow-up | 18/36 | 50 (33, 67) | 31/40 | 78 (62, 89) |

**Supplementary Table 2: Breakdown of the intervention costs**

|  | **CAU** | **SIP SMART + CAU** |
| --- | --- | --- |
| Clinical nurse specialist | £19 | £19 |
| Dietitian | £66 | £66 |
| Speech & language therapist | £58 | £148 |
| X-ray |  | £242 |
| **Total intervention cost** | **£143** | **£475** |

**Supplementary Table 3: Trajectory of EQ-5D-5L index score**

| **EQ5D-5L** | **Baseline (n = 65)** | | **4 weeks (n = 65)** | | **12 weeks** | | **24 weeks** | |
| --- | --- | --- | --- | --- | --- | --- | --- | --- |
|  | **n** | **mean (SD)** | **n** | **mean (SD)** | **n** | **mean (SD)** | **n** | **mean (SD)** |
| CAU | 40 | 0.76 (0.20) | 33 | 0.68 (0.20) | 33 | 0.71 (0.22) | 32 | 0.68 (0.22) |
| SIP SMART | 25 | 0.74 (0.21) | 23 | 0.67 (0.21) | 21 | 0.71 (0.24) | 18 | 0.68 (0.31) |

**Supplementary Table 4: Trajectories of costs**

|  | 4 weeks (n = 52) | | 12 weeks (n = 55) | | 24 weeks (n = 48) | |
| --- | --- | --- | --- | --- | --- | --- |
|  | CAU | SIP SMART | CAU | SIP SMART | CAU | SIP SMART |
|  | Cost (£) Mean (SD) | Cost (£) Mean (SD) | Cost (£) Mean (SD) | Cost (£) Mean (SD) | Cost (£) Mean (SD) | Cost (£) Mean (SD) |
| Working day lost (per week) | 265 (373) | 189 (311) | 282 (342) | 159 (304) | 224 (353) | 132 (318) |
| Supporter's working day lost (per week) | 51 (156) | 60 (173) | 51 (159) | 53 (193) | 24 (151) | 23 (115) |
| Support from other organisation (hours per week) | 20 (126) | 10 (47) | 0 (0) | 0 (0) | 3 (21) | 0(0) |
| Hospital inpatient (past 3 months) | 3310 (13364) | 297 (620) | 471 (1433) | 659 (1879) | 471 (2486) | 55 (236) |
| Hospital outpatient (past 3 months) | 2660 (2326) | 762 (1236) | 1125 (1126) | 878 (1017) | 968 (995) | 502 (851) |
| Hospital tests (past 3 months) | 1074 (3950) | 837 (3906) | 253 (550) | 81 (249) | 319 (422) | 77 (199) |
| Community and primary care (past 3 months) | 50 (87) | 74 (151) | 79 (133) | 45 (97) | 51 (87) | 45 (153) |
| Travel (past 3 months) | 380 (490) | 80 (217) | 87 (123) | 28 (62) | 61 (93) | 19 (54) |
| Total healthcare costs (past 3 months) | 7094 (15027) | 1971 (4497) | 1927 (2092) | 1664 (2674) | 1809 (2869) | 679 (989) |
| Total societal costs (past 3 months) | 8818 (15696) | 3089 (5025) | 3347 (2808) | 2538 (3784) | 2876 (3745) | 1318 (2135) |

**Supplementary Table 5: Unit costs**

|  | **Unit cost** | **Note** | **Reference** |
| --- | --- | --- | --- |
| Working day lost | £136 | Median weekly earnings were estimated at £682 for total earnings in 2023. We assume 5 working days per week. | [ONS](https://www.ons.gov.uk/employmentandlabourmarket/peopleinwork/earningsandworkinghours/bulletins/annualsurveyofhoursandearnings/2023#:~:text=Median%20weekly%20earnings%20for%20full%2Dtime%20men%20increased%20by%206.1,%C2%A3234%20to%20%C2%A3246.) |
| Supporter's working day lost | £136 | Median weekly earnings were estimated at £682 for total earnings in 2023. We assume 5 working days per week. | [ONS](https://www.ons.gov.uk/employmentandlabourmarket/peopleinwork/earningsandworkinghours/bulletins/annualsurveyofhoursandearnings/2023#:~:text=Median%20weekly%20earnings%20for%20full%2Dtime%20men%20increased%20by%206.1,%C2%A3234%20to%20%C2%A3246.) |
| Support from another organisation (hour) | £53 | Social worker (adult services) per hour with qualifications | PSSRU 2023 |
| **Hospital inpatient** | | | |
| Inpatient stay (night) | £242 | Regular day or night admission | NHS Reference Costs FY22-23 |
| Unscheduled hospital attendance | £775 | Non-Elective Inpatient - Short Stay | NHS Reference Costs FY22-23 |
| **Outpatient** | | | |
| Outpatient - Head and neck surgeon |  |  |  |
| Phone/video | £139 | WF01C - Non-Admitted Non_Face-to-Face Attendance, Follow-up (General Surgery Service) | NHS Reference Costs FY22-23 |
| Clinic | £183 | WF01A - Non-Admitted Face-to-Face Attendance, Follow-up (General Surgery Service) | NHS Reference Costs FY22-23 |
| Outpatient - Oncologist |  |  |  |
| Phone/video | £86 | WF01C - Non-Admitted Non_Face-to-Face Attendance, Follow-up (Clinical Oncology Service) | NHS Reference Costs FY22-23 |
| Clinic | £116 | WF01A - Non-Admitted Face-to-Face Attendance, Follow-up (Clinical Oncology Service) | NHS Reference Costs FY22-23 |
| Outpatient - Clinical nurse specialist |  |  |  |
| Phone/video | £58 | Cost per hour including qualifications for a hospital-based nurse at Band 6. | PSSRU 2023 |
| Clinic | £58 | Cost per hour including qualifications for a hospital-based nurse at Band 6. | PSSRU 2023 |
| Outpatient - Dietitian |  |  |  |
| Phone/video | £174 | WF01C - Non-Admitted Non_Face-to-Face Attendance, Follow-up (Dietitics Service) | NHS Reference Costs FY22-23 |
| Clinic | £197 | WF01A - Non-Admitted Face-to-Face Attendance, Follow-up (Dietitics Service) | NHS Reference Costs FY22-23 |
| Outpatient - Speech & language therapist |  |  |  |
| Phone/video | £158 | WF01C - Non-Admitted Non_Face-to-Face Attendance, Follow-up (SLT Service) | NHS Reference Costs FY22-23 |
| Clinic | £173 | WF01A - Non-Admitted Face-to-Face Attendance, Follow-up (SLT Service) | NHS Reference Costs FY22-23 |
| Outpatient - Other |  |  |  |
| Phone/video | £222 | Outpatient Procedures | NHS Reference Costs FY22-23 |
| Clinic | £222 | Outpatient Procedures | NHS Reference Costs FY22-23 |
| **Hospital tests** | | | |
| X-ray | £242 | RD32Z - Contrast Fluoroscopy Procedures with duration of more than 40 minutes | NHS Reference Costs FY22-23 |
| CT scan | £157 | RD22Z - Computerised Tomography Scan of One Area, with Pre- and Post-Contrast | NHS Reference Costs FY22-23 |
| MRI scan | £257 | RD03Z - Magnetic Resonance Imaging Scan of One Area, with Pre- and Post-Contrast | NHS Reference Costs FY22-23 |
| **Community and primary care services** | | | |
| GP surgery, doctor |  |  |  |
| Clinic | £49 | Unit cost of a GP with qualification costs, including direct care staff costs | PSSRU 2023 |
| Home visit | £49 | Unit cost of a GP with qualification costs, including direct care staff costs | PSSRU 2023 |
| Phone | £20 | Cost per intervention for a GP-led telephone triage | PSSRU 2023 |
| GP surgery, nurse |  |  |  |
| Clinic | £9 | Unit cost per hour for GP nurse with qualifications is £53. We assume that each consultation lasts 10 mins. | PSSRU 2023 |
| Home visit | £9 | Unit cost per hour for GP nurse with qualifications is £53. We assume that each consultation lasts 10 mins. | PSSRU 2023 |
| Phone | £9 | Cost per intervention for a nurse-led telephone triage | PSSRU 2023 |
| NHS 24 |  |  |  |
| Clinic | £44 | Average cost for all initial primary care actions in response to an e-consultation | PSSRU 2023 |
| Home visit | £44 | Average cost for all initial primary care actions in response to an e-consultation | PSSRU 2023 |
| Phone | £44 | Average cost for all initial primary care actions in response to an e-consultation | PSSRU 2023 |
| District nurse |  |  |  |
| Clinic | £11 | Cost per hour including qualifications for a nurse at Band 6 is £64. We assume that each consultation lasts 10 mins. | PSSRU 2023 |
| Home visit | £11 | Cost per hour including qualifications for a nurse at Band 6 is £64. We assume that each consultation lasts 10 mins. | PSSRU 2023 |
| Other nurse |  |  |  |
| Clinic | £11 | Cost per hour including qualifications for a nurse at Band 6 is £64. We assume that each consultation lasts 10 mins. | PSSRU 2023 |
| Home visit | £11 | Cost per hour including qualifications for a nurse at Band 6 is £64. We assume that each consultation lasts 10 mins. | PSSRU 2023 |
| Phone | £11 | Cost per hour including qualifications for a nurse at Band 6 is £64. We assume that each consultation lasts 10 mins. | PSSRU 2023 |
| Psychologist |  |  |  |
| Clinic | £63 | Costs per hour for a community-based staff at Bad 7 is £63. We assume that each session lasts an hour. | PSSRU 2023 |
| Home visit | £63 | Costs per hour for a community-based staff at Bad 7 is £63. We assume that each session lasts an hour. | PSSRU 2023 |
| Phone | £63 | Costs per hour for a community-based staff at Bad 7 is £63. We assume that each session lasts an hour. | PSSRU 2023 |
| Physiotherapist |  |  |  |
| Clinic | £63 | Costs per hour for a community-based staff at Bad 7 is £63. We assume that each session lasts an hour. | PSSRU 2023 |
| Home visit | £63 | Costs per hour for a community-based staff at Bad 7 is £63. We assume that each session lasts an hour. | PSSRU 2023 |
| Phone | £63 | Costs per hour for a community-based staff at Bad 7 is £63. We assume that each session lasts an hour. | PSSRU 2023 |
| **Travel** | | | |
| Travel per mile | £0.45 | Approved mileage rates from tax year 2011 to present date. | [GOV.UK](https://www.gov.uk/government/publications/rates-and-allowances-travel-mileage-and-fuel-allowances/travel-mileage-and-fuel-rates-and-allowances) |
