# Supplementary material for: Swallowing prehabilitation for people with head and neck cancer: a pilot cluster-randomised feasibility trial of the SIP SMART intervention
Source: BMJ Open. 2025 Sep 25;15(9):e103559. doi: 10.1136/bmjopen-2025-103559 (PMC12481345; doi:10.1136/bmjopen-2025-103559)
Supplement: online supplemental file 2 [file bmjopen-15-9-s002.docx]

**Supplementary Table 6: Preliminary Health Economics**

|  | **NHS perspective** | | **Societal perspective** | |
| --- | --- | --- | --- | --- |
|  | **CAU** | **CAU + SIP SMART** | **CAU** | **CAU + SIP SMART** |
| Intervention cost | £143 | £475 | £143 | £475 |
| Health service cost | £10,830 | £4,314 | £10,830 | £4,314 |
| Travel cost |  |  | £528 | £127 |
| Support from family, friends or other organisation |  |  | £596 | £585 |
| Productivity loss |  |  | £3,087 | £1,919 |
| Total cost | £10,973 | £4,789 | £15,184 | £7,420 |
| Incremental cost |  | -£6,184 |  | -£7,764 |
| 95% CI |  | (-£11,899, -£470) |  | (-£14,255, -£1273) |
| QALY (EQ-5D-5L) | 0.34 | 0.33 | 0.34 | 0.33 |
| Incremental QALY (EQ-5D-5L) |  | -0.01 |  | -0.01 |
| 95% CI |  | (-0.07, 0.05) |  | (-0.07, 0.05) |
| FACT over time | 50.31 | 52.64 | 50.31 | 52.64 |
| FACT difference over time |  | 2.33 |  | 2.33 |
| 95% CI |  | (-3.74, 8.40) |  | (-3.74, 8.40) |

**Notes:** A preliminary health economic analysis was conducted based on the complete cases (n = 27 for CAU and n=15 for SIP SMART) depicted above. This complete case analysis shows that, whilst the implementation of SIP SMART was more expensive, the overall costs to the NHS and society were lower by £6,184 (95%CI £11,899; £470) and £7,764 (95% CI £14,255; £1273) respectively. The main driver of this cost reduction was decreased health service use in hospitals for example inpatient stays for feeding tube insertions during radiotherapy treatment (see Supplementary Table 4). Differences in QALYs estimated by EQ-5D-5L were minimal but negative (-0.01). Nevertheless, QALYs estimated using FACT-HN suggested a slight improvement in the SIP SMART arm (by 2.33). We acknowledge the presence of wide confidence intervals and the nature of this pilot study which makes these results tentative. However, combined with the observation that average total costs were lower for the intervention group, this finding suggests that SIP SMART has good potential to be a dominant treatment compared to CAU. This will need to be tested in the definitive trial. The UK cancer costs questionnaire was useful to capture resource utilisation. FACT-HN appears a more nuanced measure to capture QoL for this type of intervention and the disease burden experienced by the head and neck cancer population.
