# Supplementary material for: Swallowing prehabilitation for people with head and neck cancer: a pilot cluster-randomised feasibility trial of the SIP SMART intervention
Source: BMJ Open. 2025 Sep 25;15(9):e103559. doi: 10.1136/bmjopen-2025-103559 (PMC12481345; doi:10.1136/bmjopen-2025-103559)
Supplement: Supplementary file 3 [file bmjopen-2025-103559.reviewer_comments.pdf]

PEER REVIEW HISTORY

BMJ Open publishes all reviews undertaken for accepted manuscripts. Reviewers are asked to complete a checklist review form and are provided with free text boxes to elaborate on their assessment. These free text comments are reproduced below.

ARTICLE DETAILS

Title (Provisional)

Swallowing prehabilitation for people with head and neck cancer: A pilot cluster-randomised feasibility trial of the SIP SMART intervention

Authors

Govender, Roganie; Wang, Jiunn; Marston, Louise; Pizzo, Elena; Taylor, Stuart; Nazareth, Irwin

---

VERSION 1 - REVIEW

**Reviewer**	**1**
**Name**	**Charters, Emma**
**Affiliation**	**Chris O'Brien Lifehouse**
**Date**	**17-May-2025**
**COI**	**None**

Thank you for the opportunity to review this pilot paper of the SIP SMART intervention.

Abstract

- Clearly articulated aims and outcomes of this feasibility study. excellent study design, given both randomisation and engagement of multiple sites across the NHS.

- Noted that the adherence rate was over 50%, I wonder what the authors had expected and what was their metric for success in this regard?

Introduction

- SIP SMART protocol has been documented prior, with a sound theoretical basis for the behavioural approach to the intervention proposed. The personalised intervention program based on an instrumental assessment is a refreshing deviation from a ‘one size fits all’ prehabilitation program which have, in the past, resulted in limited benefit.

- The background for and scope of this trial is well written and clear for the reader. The personalised intervention also makes for a more pragmatic trial which is likely to be more amenable to translation to clinical practice in the future should it be effective.

Method

- Did any of the sites recruited have a standard of care which was more like SIP SMART, than the care as usual?

- Heterogenous sample, benefit is, as stated above, that it is a more pragmatic trial which is representative of a clinical load. Heterogenous sample using a personalised intervention program.

- At the CAU sites, if the patient presented with dysphagia did the have access to a VFSS?

- How did you assess patient adherence? Was there any planned target that you considered an acceptable level of adherence?

Discussion

- Did you consider interviewing the clinicians who were trained on their perspectives on the efficacy of a different approach to supporting patients? did those in the intervention group feel that it was a substantial shift in approach to their current practice standard?

-

**Reviewer**	**2**
**Name**	**Tanriverdi, Muberra**
**Affiliation**	**Bezmialem Vakif Universitesi, Physiotherapy and Rehabilitation**
**Date**	**22-May-2025**
**COI**	**None**

---

1. The rationale for cluster randomisation is sound (i.e., avoiding contamination), but the manuscript should include more critical reflection on the trade-offs. For instance, the implications of regional clustering (intervention sites in the north and control in the south) are noted, but not sufficiently explored in terms of possible biases or differences in standard care.

2. The manuscript indicates a substantial amount of missing follow-up data, particularly in the intervention arm. While site-specific challenges are acknowledged, the strategies to address missingness (e.g., imputation plans, mitigation in the definitive trial) are not discussed adequately.

3. Please provide more information on how missing data were handled in the statistical and economic analyses.

4. The preliminary cost-effectiveness analysis is interesting, but the interpretation of findings needs greater clarity. The SIP SMART intervention is reported as more expensive but overall costs were paradoxically lower. A clearer breakdown of these findings, including confidence

intervals around estimates and the robustness of conclusions given the small sample, is needed.

5. The statement that SIP SMART was “dominant” based on FACT-HN scores is not statistically justified. Please temper this language or qualify the conclusions appropriately.

6. The use of a traffic light system is appropriate, but the explanation of how the thresholds were set and whether they were pre-specified should be expanded.

7. Also, clarify whether these criteria were evaluated independently or in combination when deciding on the feasibility of a definitive trial.

8. Further detail is needed on how adherence to the intervention protocol was monitored beyond self-report.

9. Was any assessment made of potential contamination in the CAU sites or drift in standard care delivery over time?

10. While the manuscript is comprehensive, the density of the text, particularly in the Methods and Results sections, can be challenging. Consider reducing redundancy and using more subheadings or summary tables to improve readability.

11. Ensure consistent use of terminology (e.g., CAU vs. usual care vs. standard care).

12. Please provide a CONSORT flow diagram adapted for cluster trials.

13. Consider including a table summarising adverse events and their categorisation (SAE vs. AE) by arm.

14. Clarify the ethical implications of deaths not being considered SAEs unless directly related to the intervention.

15. In the Discussion, temper speculative statements about cost-effectiveness given the limited data.

**Reviewer**	**3**
**Name**	**Frowen, Jacqui**
**Affiliation**	**Peter MacCallum Cancer Centre, Nutrition & Speech Pathology**
**Date**	**28-May-2025**
**COI**	**None**

Thank you for the opportunity to review this manuscript, which describes the feasibility of delivering a swallowing prehabilitation intervention (SIP SMART) in a two-arm cluster randomised pilot trial design.

The manuscript is very clear and well written. It addresses an important issue that is highly relevant in both clinical care and research, and I commend the authors for taking such a systematic approach

to investigating the intervention. Hopefully this approach will ensure timely implementation into routine care, should effectiveness be confirmed.

Please see specific comments below.

1. The ‘broadly consistent’ elements of CAU at all sites is addressed (p12, line 56 onwards), and it has been clarified that, following treatment, all patients followed usual care re: rehabilitation. However, presumably many patients at the CAU centres ran into problems during radiotherapy and needed additional SLT input, above and beyond the single baseline assessment/education? Was this data collected as this will need to be controlled for in a future trial (including whether practice differed by cluster/centre)?

2. Please review the wording of Objective 2, p10, line 8. This objective states that ‘consent’ relates only to patients agreeing to provide outcome data, which is different to agreeing to participate in an intervention trial. This implies that patients had no choice but to receive either CAU or the intervention, and their consent only related to whether they provided outcomes data or not. All other elements of the paper (from Methods onwards) suggest the feasibility data collected related to recruitment to the study.

3. Methods – recruitment of sites: please clarify (if this is indeed correct) that the sites were relatively consistent in their treatment regimens for specific H&N tumour sites and sizes. While this may be consistent across all NHS sites in the UK (as it is in Australia), this requires clarification.

4. P12 line 24 says “SIP SMART2 study” I assume the 2 is a typo and not a modified version of the SIP SMART intervention? Although I see this term is also used in figure 1. Please clarify.

5. Table 1: please review the symbols/spacing used in the final row for Go and Amend – Go reads as “70% ‘and also’ for MDADI” (use ‘greater than’ symbol instead of +) and for Amend please ensure a space between the dash so it’s obvious this is a range.

6. P17 line 3 – please clarify if this refers to recruitment of the centres or recruitment of participants at each centre.

7. Figure 2 – it looks like there is some obscured text in the blue Cluster Enrolment box

8. Given the SIP SMART intervention relies on clinicians utilising behaviour change techniques, was clinician adherence to the SIP SMART intervention assessed or monitored?

9. The clinical outcome measures used in the study are poorly defined or in some cases not defined at all. Although a detailed outcome of these measures is not required given the purpose of this particular paper, some additional detail is required. Specifically:

    a. Provide references for MDADI and FACT-HN at their first mention in text
    b. Provide references for the additional assessments outlined in Table 2 (e.g. PSS-HN, WST)
    c. Please briefly explain how the ‘length of life’ variable is determined when calculating QALY in this cohort with this intervention (p16, line 46)
    d. Table 3 and Supp table 1: please clarify what the AUDIT-C score and FIGS are – these are not mentioned anywhere else in the manuscript (including in table 2 schedule) and are not widely-known measures

e. To better understand results (acknowledging that this study is not looking at effectiveness), please state what each outcome measure (PSS-HN, FIGS, FACT-HN, MDADI, EQ5D) in table 3 is out of. This can probably be done simply in the Characteristic column, e.g. “MDADI Composite Score (out of 100)”

10. Table 4 and associated text – ICER is not defined, making these results impossible to interpret

11. One of the key feasibility findings from this study was the significant amount of missing data with very few patients followed up at one site in the intervention arm. This issue means that the intervention arm actually fell within Amber (proceed with changes) or Red (do not proceed) criteria. Given the primary purpose of this pilot trial was to test the cluster-randomised design, this issue should be addressed in more detail, including what the reasons were for the missing data (which I assume you know given qualitative data was collected for objective 6 in the flowchart) and what changes will be made to try and mitigate the issue in future studies – not just that “it will be crucial to have proactive steps for the definitive trial to address this potential problem.” ADDIT: I have now come to the final sentence of the discussion – does this relate to my comment? If so, this sentence should immediately follow the comments on p26 line 19/20, but further detail is still required in this paper.

VERSION 1 - AUTHOR RESPONSE

Reviewer 1

<u>Abstract-</u> Clearly articulated aims and outcomes of this feasibility study. excellent study design, given both randomisation and engagement of multiple sites across the NHS. - Noted that the adherence rate was over 50%, I wonder what the authors had expected and what was their metric for success in this regard?

Based on our preliminary work, we anticipated that adherence would be around 50% when measured using self-reports as part of the *self-monitoring behaviour change technique*. Anything over 50% (undertaking more than 50% of the exercises provided for more than 50% of the time window) was viewed as good success. This is further discussed in the manuscript (page 24)

<u>Introduction-</u> SIP SMART protocol has been documented prior, with a sound theoretical basis for the behavioural approach to the intervention proposed. The personalised intervention program based on an instrumental assessment is a refreshing deviation from a ‘one size fits all’ prehabilitation program which have, in the past, resulted in limited benefit. The background for and scope of this trial is well written and clear for the reader. The personalised intervention also makes for a more pragmatic trial which is likely to be more amenable to translation to clinical practice in the future should it be effective.

Thank you for this positive feedback.

<u>Method-</u> Did any of the sites recruited have a standard of care which was more like SIP SMART, than the care as usual?- Heterogenous sample, benefit is, as stated above, that it is a more pragmatic trial which is representative of a clinical load. Heterogenous sample using a personalised intervention program.- At the CAU sites, if the patient presented with dysphagia

did they have access to a VFSS?- How did you assess patient adherence? Was there any planned target that you considered an acceptable level of adherence?

As this was a pragmatic trial, if a patient at the CAU site was referred for a VFSS for clinical reasons, access was as per the site's clinical protocol. This was not considered to interfere with how the VFSS was used in the SIP SMART intervention.

Please see above for information on adherence.

Discussion- Did you consider interviewing the clinicians who were trained on their perspectives on the efficacy of a different approach to supporting patients? did those in the intervention group feel that it was a substantial shift in approach to their current practice standard?

Yes, focus groups with clinicians captured this information which will be discussed in our forthcoming qualitative paper

Reviewer: 2

1. The rationale for cluster randomisation is sound (i.e., avoiding contamination), but the manuscript should include more critical reflection on the trade-offs. For instance, the implications of regional clustering (intervention sites in the north and control in the south) are noted, but not sufficiently explored in terms of possible biases or differences in standard care.

Thank you for this observation. We have added a sentence to the Limitations section and re-ordered the text to improve clarity.

2. The manuscript indicates a substantial amount of missing follow-up data, particularly in the intervention arm. While site-specific challenges are acknowledged, the strategies to address missingness (e.g., imputation plans, mitigation in the definitive trial) are not discussed adequately.

Thank you for pointing this out - We have added the below text to the section on key learning

"To reduce the amount of missing data, the larger trial with increased funding could employ strategies such as online questionnaires with automatic reminders, incentivisation such as shopping vouchers for completion of all measures as well as more staff resources dedicated to collection of outcomes. In a definitive trial it will also be appropriate to consider statistical or imputation methods for handling missing data "

3. Please provide more information on how missing data were handled in the statistical and economic analyses.

Thank you for the opportunity to clarify. We present complete case analyses in this paper. Given the statistical analysis was descriptive and one of the aims of the study was to see whether data could be collected, complete case analysis is appropriate. We did a predictors of missingness analysis (as described in the methods) for MDADI and FACT-HN, with the only significant predictor of missingness being site.

4. The preliminary cost-effectiveness analysis is interesting, but the interpretation of findings needs greater clarity. The SIP SMART intervention is reported as more expensive but overall costs were paradoxically lower. A clearer breakdown of these findings, including confidence

intervals around estimates and the robustness of conclusions given the small sample, is needed.

Thank you for your suggestions. We've updated the table, so that differences in each cost component between groups can now be easily observed. The overall costs were lower for the treatment group due to a lower incidence of hospital admissions, as shown in the Supplementary Table 4. We have also included 95% confidence intervals for incremental costs and outcomes, as suggested. An update in the methodology has been provided as well.

5. The statement that SIP SMART was "dominant" based on FACT-HN scores is not statistically justified. Please temper this language or qualify the conclusions appropriately.

Thank you for your suggestion. The term "dominant" is commonly used in health economics to describe a case where costs are lower and outcomes are better on average. We understand that this term may be misleading for some readers, so we have clarified that uncertainty remains, even though the treatment is dominant on average.

6. The use of a traffic light system is appropriate, but the explanation of how the thresholds were set and whether they were pre-specified should be expanded

As per Table 1, the criteria were set apriori based on the key feasibility outcomes. The thresholds were determined by the trial management group using information from the literature and knowledge of the practical context.

7. Also, clarify whether these criteria were evaluated independently or in combination when deciding on the feasibility of a definitive trial.

Thank you for highlighting this – we have added a sentence to clarify.

8. Further detail is needed on how adherence to the intervention protocol was monitored beyond self-report.

We have added the below to the Methods Section.

> *"Each SLT delivering the intervention was required to audio record at least one consultation which would allow for fidelity assessment, whilst patient adherence was reported using a study specific self-reported questionnaire"*

9. Was any assessment made of potential contamination in the CAU sites or drift in standard care delivery over time?

Based on reviewing a sample of recordings of consultations from both arms and from peri-trial focus groups, key elements of the intervention were preserved in the intervention group and did not appear to cross into the CAU group. This aspect will be further explored in our accompanying qualitative paper.

10. While the manuscript is comprehensive, the density of the text, particularly in the Methods and Results sections, can be challenging. Consider reducing redundancy and using more subheadings or summary tables to improve readability.

Thank you – The Methods and Results already contain subheadings in bold text. We also already have the maximum number of summary Tables. See response to Q12 below as well. We realise that there is a lot to report and have therefore tried to separate the more qualitative results and reserved these for a separate paper.

11. Ensure consistent use of terminology (e.g., CAU vs. usual care vs. standard care).

We have used CAU consistently in referring to the CAU trial arm. We use usual care as a more general term in the text, when not specifically referring to the CAU trial arm.

12. Please provide a CONSORT flow diagram adapted for cluster trials.

This has been provided. Apologies if this did not download with your review documents. Please note that Figures1 (participant recruitment) and Figure 2 (CONSORT Flowchart) are attached, as per journal guidelines, after appendices and references and not placed within the manuscript text.

13. Consider including a table summarising adverse events and their categorisation (SAE vs. AE)by arm.

We already have the maximum Tables, however we have added more detail here.

14. Clarify the ethical implications of deaths not being considered SAEs unless directly related to the intervention.

We have revised this sentence to make it clearer.

15. In the Discussion, temper speculative statements about cost-effectiveness given the limited data.

Thank you for your suggestion. We've adjusted the statements regarding the health economic findings by addressing the uncertainty around the estimates

Reviewer: 3

Thank you for the opportunity to review this manuscript, which describes the feasibility of delivering a swallowing prehabilitation intervention (SIP SMART) in a two-arm cluster randomised pilot trial design. The manuscript is very clear and well written. It addresses an important issue that is highly relevant in both clinical care and research, and I commend the authors for taking such a systematic approach to investigating the intervention. Hopefully this approach will ensure timely implementation into routine care, should effectiveness be confirmed. Please see specific comments below.

1. The 'broadly consistent 'elements of CAU at all sites is addressed (p12, line 56 onwards), and it has been clarified that, following treatment, all patients followed usual care re: rehabilitation. However, presumably many patients at the CAU centres ran into problems during radiotherapy and needed additional SLT input, above and beyond the single baseline assessment/education? Was this data collected as this will need to be controlled for in a future trial (including whether practice differed by cluster/centre)?

Thank you. SIP SMART is intended to be a prehabilitation consultation in addition to standard care and therefore did not preclude patients receiving what is usually provided. Yes, the number of SLT sessions was collected in both arms (methods section p.) Focus group discussions also provided an opportunity to reflect on any practice variation. This will be further explored in our forthcoming qualitative paper.

2. Please review the wording of Objective 2, p10, line 8. These objective states that 'consent 'relates only to patients agreeing to provide outcome data, which is different to agreeing to participate in an intervention trial. This implies that patients had no choice but to receive either CAU or the intervention, and their consent only related to whether they provided outcomes data or not. All other elements of the paper (from Methods onwards) suggest the feasibility data collected related to recruitment to the study.

Thank you – we have inserted "who consent to participation"

3. Methods – recruitment of sites: please clarify (if this is indeed correct) that the sites were relatively consistent in their treatment regimens for specific H&N tumour sites and sizes. While this may be consistent across all NHS sites in the UK (as it is in Australia), this requires clarification.

Thank you – we have added a sentence clarifying this under the Participants subheading

4. P12 line 24 says "SIP SMART2 study" I assume the 2 is a typo and not a modified version of the SIP SMART intervention? Although I see this term is also used in figure 1. Please clarify.

SIP SMART is the intervention, SIP SMART2 (ISRCTN12377415) refers to this current pilot study to differentiate it from the previous preliminary study.

5. Table 1: please review the symbols/spacing used in the final row for Go and Amend – Go reads as "70% 'and also 'for MDADI" (use 'greater than 'symbol instead of +) and for Amend Amend please ensure a space between the dash so it's obvious this is a range.

Done

6. P17 line 3 – please clarify if this refers to recruitment of the centres or recruitment of participants at each centre.
This refers to recruitment of participants – added to text.

7. Figure 2 – it looks like there is some obscured text in the blue Cluster Enrolment box
Thank you, this has been rectified.

8. Given the SIP SMART intervention relies on clinicians utilising behaviour change techniques, was clinician adherence to the SIP SMART intervention assessed or monitored?
Yes, clinicians delivering the intervention were provided with a manual and prompt checklist and were each asked to make at least one recording of a consultation with participant consent. Adherence to the key aspects of the intervention were rated from the recordings using a study-specific checklist that incorporated the main behaviour change techniques. Again, this will be discussed separately.

9. The clinical outcome measures used in the study are poorly defined or in some cases not defined at all. Although a detailed outcome of these measures is not required given the purpose of this particular paper, some additional detail is required. Specifically: a. Provide references for

MDADI and FACT-HN at their first mention in text. Provide references for the additional assessments outlined in Table 2 (e.g. PSS-HN, WST) c. Please briefly explain how the ‘length of life ‘variable is determined when calculating QALY in this cohort with this intervention (p16, line 46)d. Table 3 and Supp table 1: please clarify what the AUDIT-C score and FIGS are – these are not mentioned anywhere else in the manuscript (including in table 2 schedule) and are not widely-known measures. To better understand results (acknowledging that this study is not looking at effectiveness), please state what each outcome measure (PSS-HN, FIGS, FACT-HN, MDADI, EQ5D) in table 3 is out of. This can probably be done simply in the Characteristic column, e.g. “MDADI Composite Score (out of 100)”

Thank you for bringing this to our attention. The measures used have all been described in our earlier protocol paper, however we have added a brief explanation, and score range, and references in Tables 2 and 3 for easy access.

10. Table 4 and associated text – ICER is not defined, making these results impossible to interpret

Thank you – Based on suggestions from the editor, we have removed this aspect of the health economics as it is too preliminary at this stage. Instead, we have presented the costs analysis.

11. One of the key feasibility findings from this study was the significant amount of missing data with very few patients followed up at one site in the intervention arm. This issue means that the intervention arm fell within Amber (proceed with changes) or Red (do not proceed) criteria. Given the primary purpose of this pilot trial was to test the cluster-randomised design, this issue should be addressed in more detail, including what the reasons were for the missing data (which I assume you know given qualitative data was collected for objective 6 in the flowchart) and what changes will be made to try and mitigate the issue in future studies – not just that “it will be crucial to have proactive steps for the definitive trial to address this potential problem.” ADDIT: I have now come to the final sentence of the discussion – does this relate to my comment? If so, this sentence should immediately follow the comments on p26 line 19/20, but further detail is still required in this paper

Thank you. We have now addressed this in combination with suggestions from reviewer 2

VERSION 2 - REVIEW

**Reviewer**	**3**
**Name**	**Frowen, Jacqui**
**Affiliation**	**Peter MacCallum Cancer Centre, Nutrition & Speech Pathology**
**Date**	**05-Aug-2025**
**COI**	

Thank you for addressing the comments by all reviewers and editor. Two minor comments:

- re: my comment #4; thank you for clarifying that SIP SMART is the intervention whereas SIP SMART2 refers to this current pilot study. Although the words "SIP SMART2 study" have been added to the trial registration number, most readers will miss this and might still cause confusion. Please clarify in the main text - for example, in the Introduction: "The primary aim of the current external pilot trial (named “SIP SMART2”) was...."

- re: my comment #7; a copy of Figure 2 was not available for me to review - I trust the editors are able to check that the obscured text in the blue Cluster Enrolment box has been fixed.

VERSION 2 - AUTHOR RESPONSE

Reviewer 3:

Thank you for addressing the comments by all reviewers and editor. Two minor comments:

- re: my comment #4; thank you for clarifying that SIP SMART is the intervention whereas SIP SMART2 refers to this current pilot study. Although the words "SIP SMART2 study" have been added to the trial registration number, most readers will miss this and might still cause confusion. Please clarify in the main text - for example, in the Introduction: "The primary aim of the current external pilot trial (named “SIP SMART2”) was...."

Thank you – for clarity we have now incorporated this into the abstract and added the information in the introduction as you illustrate above.

- re: my comment #7; a copy of Figure 2 was not available for me to review - I trust the editors are able to check that the obscured text in the blue Cluster Enrolment box has been fixed.

I confirm that this has been fixed.
